# Sustainable Service-Learning in Physical Education Teacher Education: Examining Postural Control to Promote ASD Children’s Well-Being

**DOI:** 10.3390/ijerph18105216

**Published:** 2021-05-14

**Authors:** Teresa Valverde-Esteve, Celina Salvador-Garcia, Jesús Gil-Gómez, María Maravé-Vivas

**Affiliations:** 1 Department of Didactics of Music, Visual and Body Expression, University of Valencia, 46022 Valencia, Spain; Teresa.Valverde@uv.es; 2 Faculty of Education, Universidad Internacional de la Rioja, Avenida de la Paz, 137, 26006 Logroño, Spain; 3 Department of Education and Specific Didactics, Faculty of Humanities and Social Sciences, University Jaume I, 12071 Castellón, Spain; jegil@uji.es (J.G.-G.); marave@uji.es (M.M.-V.)

**Keywords:** service learning, physical education teacher education, Autism Spectrum Disorder, physical education, postural control

## Abstract

As classrooms become more and more diverse, it is imperative to provide physical education teacher education (PETE) students with opportunities to develop competencies that promote quality education for all students. In this study, PETE students applied a physical education service-learning (SL) program aimed at enhancing Autism Spectrum Disorder (ASD) children’s motor domain and general well-being—objectives that are connected to the third focus of the United Nations’ Sustainable Development Goals (SDGs). Traditionally, research on SL has focused on students’ outcomes, and there is a call to examine SL’s effects on service receivers, which is the gap this paper aspires to fill. The aim of this study was to measure the postural control of children with ASD who were involved in a 6-month SL program in comparison to ASD peers in a control group. A quasi-experimental design was used in which a total of 29 children with ASD participated. The results of the experimental group showed a significant improvement in the vestibular pathways, an improvement trend in the somatosensorial and visual pathways and improvements in the dynamic tests. This study provides valuable feedback about how SL programs can benefit ASD children to improve their postural control, thus contributing to the third SDG concerned with well-being promotion.

## 1. Introduction

As classrooms become more and more diverse, it is imperative for teachers to offer appropriate responses that ensure high-quality education and attend to the needs of all students. This is in line with the United Nations 2030 Agenda for Sustainable Development, particularly with its fourth goal: quality education. For this to be achieved, teacher training is of utmost importance. At this educational stage, pre-service teachers should learn content and develop competencies and attitudes which are basic to attend to diversity. In the field of physical education teacher education (PETE), learning should consider how to attend to children with special education needs, because some studies have found that physical education (PE) teachers show a lack of perceived competence to include them successfully in lessons [1,2]. For example, Tant and Watelain [1] carried out a systematic review on inclusion in PE, and they state that pre-service training is one of the most relevant factors influencing the inclusion of students with disabilities. As a consequence, university professors should provide pre-service teachers with didactic opportunities to enhance the skills and attitudes that help them to properly attend to students with educational needs (SEN); furthermore, using methodological approaches that let university students be in direct contact with SEN children may be an effective strategy to acquire such skills.

At this point, service learning (SL), an active and participative methodology, emerges as an interesting possibility to consider, since it is a student-led approach through which learners have to face a social problem while developing their competencies [3]. Reviews on SL in the field of PE and applied with SEN justify its usefulness to enhance teaching competencies in pre-service teacher training [4,5]. It is interesting to note, though, that studies in this arena have traditionally been focused on analyzing the effects of SL on service providers, and thus service receivers have usually been neglected. As a result, there is a call for new investigations aimed at examining the effects of SL on community participants [6,7]). This is precisely the niche that the present investigation seeks to address since it is focused on exploring the impact of SL on children receiving the service. Particularly, among the different SEN possibilities that exist, the study is focused on children with Autism Spectrum Disorder (ASD) because of its high prevalence of 18.5 per 1.000 [8]. Therefore, there is a need to improve teacher training to properly include this group of students in the lessons.

Individuals with ASD show an altered capacity to interact and communicate [9,10,11,12], in terms of frequency, type and quality of communication [13]. This results in a lack of social skills. Furthermore, research has shown that individuals with ASD have difficulties processing and modulating sensory inputs [14,15,16,17,18]. Specifically, they show hypo or hyperreactivity or unusual interest in sensory aspects of movement [19], poor motor coordination and motivation [20], difficulty self-monitoring [21] and altered postural control in bipedal stance compared to their typically developing (TD) peers [22]. In general, children with ASD may have a risk of motor skill delay and motor difficulty, altered manual skills, ball skills and static and dynamic balance when compared to TD children [23]. According to the literature, ASD children have reduced opportunities to participate in extracurricular PE activities [24]. Therefore, their possibilities of developing motor skills are substantially hampered. As a consequence, it is necessary to develop physical activity (PA) programs and activities aimed at ASD children, such as the proposal carried out for this research to provide them with playful opportunities, letting them develop motor skills and taking advantage of the social and personal benefits underlying PA practice. PA is shown to help children with ASD to increase their social skills [12,25] and motor skills, improve their use of materials (i.e., mats, hoops and balls) and elicit spontaneous interactions in standardized contexts [11] by performing activities such as walking, running and jumping. Indeed, these motor actions are particularly important for the daily living activities of children with ASD, and thus for improving their quality of life [26]. Among other aspects, these motor skills may be especially important in enhancing the child’s autonomy and independence in daily living [26,27].

Postural control is a fundamental skill that is required to maintain postural orientation and equilibrium. It requires active control and alignment according to gravity, support surface, visual environment and afferent pathways, involving the reception, processing and integration of feedback from the visual, vestibular and proprioceptive senses [28]. Therefore, postural control is conditioned by movement strategies to stabilize the center of body mass during disturbances of stability, within the safety limits that the erect position represents [29], and requires active coordination of ankle dorsi/plantar flexion and hip abduction/adduction to stabilize the upper body [30]. In addition, postural control is an integral part of all movements. Consequently, improvements in postural control should lead to improvements in all movements and are therefore closely related to quality of life and well-being [29,31], which are connected to the third Sustainable Development Goal (SDG): good health and well-being.

Postural control can be assessed through the center of pressures (CoP), which is a way of measuring one’s sense of balance [32], thus allowing for the evaluation of variations during one’s time spent in antero-posterior (AP) and medio-lateral (ML) trajectories. Therefore, a lower variability would be the result of better postural control [33]. Measuring postural control is a process of quantitative data acquisition using force platforms. Previous studies have addressed the measurement of postural control in children with ASD [34,35,36]. Specifically, most of them focused on comparing the balance in children with ASD to TD children. However, to our knowledge, no studies have reported the effect of a 6-month extracurricular PE program based on perceptual-motor skills comparing two groups of ASD children.

Appropriate PE interventions may contribute to an overall improvement in motor skills or even particular aspects such as postural control. However, interventions aiming to provide ASD children with opportunities to engage in PA are scarce and, to our knowledge, no studies have been carried out in a Spanish context. ASD children have reduced postural control when compared with TD children [23]. This is especially reflected in their unreliability in using visual or vestibular information [37] and tendency to perform a greater general sway area [38,39], specifically in the AP and lower ML direction [22,40,41,42]. Moreover, children with ASD have fewer opportunities to engage in PA outside of school [24]. Therefore, there is a need to discover whether providing this population with appropriate PE activities can help them improve their motor skills, particularly postural control, because of its relationship with all movements and its importance in guaranteeing a greater quality of life and well-being in ASD children [43].

In a study by Kim et al. [35], the authors reported significant improvements after 16 sessions of taekwondo intervention. In the same line, a study by Giagazoglou et al. [44] reported significant improvements in the CoP of AP and ML trajectories with opened and closed eyes (*p* < 0.05) after the performance of a trampoline exercise intervention of 12 weeks. However, the study by Cheldavi et al. [34] did not report significant differences in the pre and post-tests for the hard and foam surface conditions, with eyes opened and closed. The authors only found improvements when comparing the results among the experimental and control groups in ML trajectories (*p* = 0.007) and velocity in the visual conditions (opened and closed) (*p* = 0.009).

Therefore, in an attempt to continue unveiling these aspects, the aim of this study was to investigate the changes in static and dynamic postural control experienced by children with ASD who undertook a 6-month PE program (ASD_E_) and to compare them to a group of ASD children who did not participate in the program (ASD_C_). Another objective was to assess the relative contribution of visual, somatosensory and vestibular inputs. Our hypotheses were that (1) ASD_E_ would show significant improvements in terms of static and dynamic postural control, (2) ASD_E_ would show significantly higher CoP velocities when their evolution was compared to that of the control group after the 6-month intervention program, and (3) the relative contributions of the afferent pathways would improve in ASD_E_ after the intervention program.

## 2. Materials and Methods

### 2.1. Design

The study used a quasi-experimental design of 2 non-equivalent groups (experimental and control), with pre-test and post-test measures, to compare how participation in the intervention program affected the participating ASD children. This design makes it possible to ascertain the causal relationship (effect) of the independent variable (SL program) on the dependent variable (postural control) [45].

### 2.2. Sample

**Figure 1:**
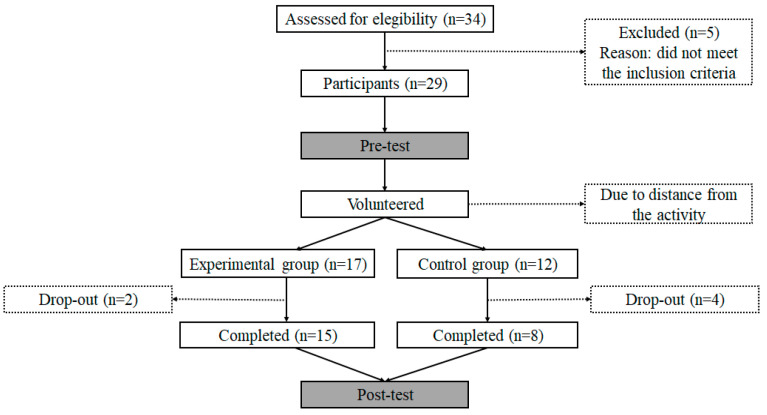
Flow chart of the inclusion process.

Participants were recruited within a 100 km radius of the university where the research was conducted. Initially, 34 children with ASD volunteered for this activity; however, only 29 participated (see Figure 1). As the recent literature review by Reinders et al. [46] shows, studies with ASD populations usually rely on data gathered with a similar number of participants to our study. The families of the participating children could decide whether to be included in the experimental group or the control group so that everyone had the opportunity to take part in the program. Some ASD children did not complete the post-test. Therefore, the final sample was formed by 23 children, divided into the experimental group (ASD_E_: n = 15 children; 11 boys, 4 girls; age: 10.43 ± 3 years old; body mass: 41.52 ± 14.65 kg; height: 137.47 ± 13.94 cm) and the control group (ASD_C_: n = 8 children; 7 boys, 1 girl; age: 10.13 ± 3.09 years old; body mass: 37.2 ± 16.0 kg; height: 140.13 ± 17.18 cm). The groups were previously compared in order to verify that they were not significantly different (age: *p* = 0.809, height: *p* = 0.692, body mass: *p* = 0.189).

The families of the children involved were informed about the research and the criteria for participating. The inclusion criteria to participate in the study consisted of (1) a clinical diagnosis (made by trained professionals) based on the diagnostic standards for ASD established in the DSM-4 or DSM-5, (2) an age between 5 and 16 years old, (3) the absence of illnesses that reduce their ability to engage in physical activity, (4) an IQ ≥ 70 and (5) the ability to follow instructions.

### 2.3. Materials

All participants completed the balance tests using the Basic Balance Master system force platform (NeuroCom, version 9.2., Clackamas, OR, USA). This platform, through its software, calculates variables related to the CoP at a sampling rate of 100 Hz, the percentage of weight-bearing (angle; º) on each side and the balance velocity (º/s). We used the velocity magnitudes to characterize postural control. We evaluated the squat angle with a goniometer (SAEHAN Corp., Korea). Once we had obtained these data, we entered them in Microsoft Excel (Version 10 for Windows, Microsoft) and exported them to SPSS V. 26 (SPSS Inc., Chicago, IL, USA).

### 2.4. Procedures

For the assessment parameters, participants carried out a static and dynamic balance test. The protocol of the static balance test consisted of performing 4 standing conditions with a codified clinical test of sensory interaction on balance (CTSIB) for 10 s, obtaining the mean of 3 trials: (1) static position on a firm surface with eyes open (EO_QS), (2) static position on a firm surface with eyes closed (EC_QS), (3) static position on a foam surface with eyes open (EO_FS), (4) static position on a foam surface with eyes closed (EC_FS). As reported by Molloy et al. [36], the main afferent input pathways involved according to the different tests are shown in Table 1. We obtained the results for the afferent pathways following criteria established in the study by Fong et al. [47] as the result from the following conditions: somatosensory (EC_QS/EO_QS), visual (EO_FS/EO_QS) and vestibular (EC_FS/EO_QS).

We also measured static unilateral balance under 4 conditions for 10 s each, obtaining the mean of 3 trials: (5) left leg with eyes open (EO_LL), (6) left leg with eyes closed (EC_LL), (7) right leg with eyes open (EO_RL), (8) right leg with eyes closed (EC_RL). 

**Figure 2:**
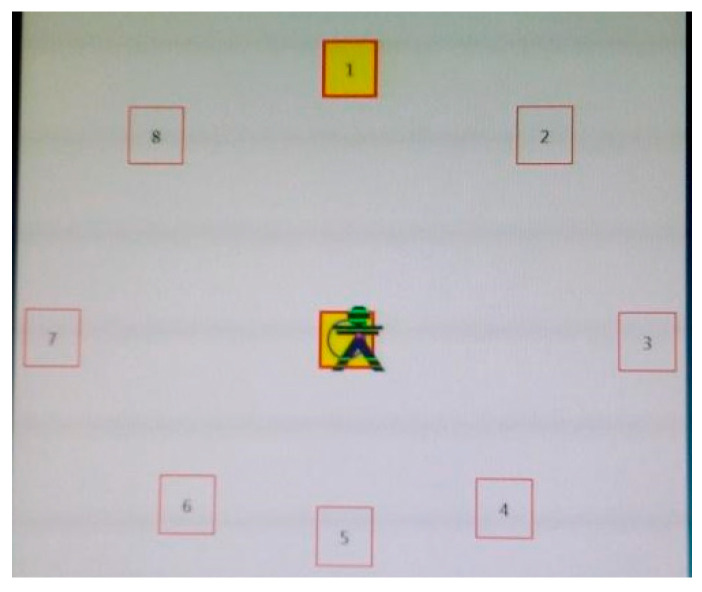
Limits of the stability test procedure.

The dynamic tests consisted of (9) the limits of stability (LOS) test, in which participants voluntarily had to shift their center of gravity towards the limit of their position in 8 pre-established directions (F: forward, RF: right-forward; R: right; RB: right-backward; B: backward; LB: left-backward; L: left; LF: left-forward) after standing for 8 s in the central position (Figure 2). This protocol was repeated starting from the center in all 8 trials. The children were asked to start moving from the center as fast as possible in the specified direction once the 8 s had finished. The software verified whether the procedures were performed correctly or whether they had to be repeated if the children had moved in any direction before the 8 s or had moved their foot position from the platform.

### 2.5. Intervention Program

The experimental group undertook the PE program twice a week for a duration of 1 h each session between November and May. The control of participants’ attendance showed a high degree of adherence to the sessions (91.2%). Each child with ASD received an invitation to take part in the PE sessions with a sibling or a friend so that they felt more comfortable.

The program was based on SL applied by PETE students, and it was designed in order to promote SDG number three (good health and well-being) and four (quality education). The objectives of the service were, on the one hand, to provide playful PA to ASD children, and, on the other hand, to promote their motor skills and well-being. Regarding the objectives for PETE students’ learning, the SL program aimed at developing the social and educational inclusion of ASD children.

PETE students worked in groups of 4–5 members and each group took care of a few children. The children-to-staff ratio was between 3 and 5 depending on the session and group. Pre-service teachers had to consider the motor skills of the children they were working with and design PE sessions to improve them. For this, they had to apply the contents covered in the curriculum of the subject. University professors were also part of the program: they supervised sessions and guided group reflections after each session since this is an integral part of SL proposals [48].

All sessions had a very simple structure that focused on providing children with ASD with opportunities for the future practice of healthy, recreational and educational PA: 5–10 min of simple warm-up activities, 45 min of activities or games focusing on motor skills and a dynamic cool-down to finish the session. Each session was composed of both group dynamics (e.g., parachute, traditional games and dancing activities) which were carried out mainly during the warm-up and cool-down parts of the session, and activities in groups of 3 or 4 children based on age ranges. These activities were specifically aimed at developing fine and gross motor coordination, balance and basic perceptual-motor skills such as running, jumping, throwing, catching and participating in basic traditional games. Most of the exercises were explained by imitation since visual instruction is more effective than verbal instruction for children with ASD [49]. The children in the control group, on the other hand, did not carry out extracurricular activities involving PA. This variable was controlled through previous interviews and subsequent reports from the families.

### 2.6. Data Analysis

From the squat test, we obtained the percentage of weight-bearing by the right and left legs (%). The modified CTSIB and unilateral tests reported magnitudes of the CoP velocity (º/s). From the LOS, we obtained the parameters of reaction time (RT) (s), movement velocity (MV), point of maximum excursion (MXE) and directional control (DCL).

### 2.7. Statistical Analysis

After the analysis of the normal distribution of the sample, we observed that the static results showed a normal distribution, whilst the dynamic tests and velocity of the CoP reported a non-normal distribution. We performed descriptive analysis, including the mean and standard deviation for the normal results and median and quartile ranges (Q1 and Q3) for the non-normal results. We also obtained Cohen’s *d* [50] (small effect: 0.2 < *d* < 0.4; medium effect: 0.5 < *d* < 0.7; large effect: *d* > 0.8). For the static conditions, we performed Student’s t-test for unpaired samples for the preconditions at a level of significance of *p* > 0.05 to verify that there were no significant differences between the groups. We also performed interval coefficients (ICC_95%_). The effects of the results within groups were assessed by ANOVAs (group x condition) and Bonferroni’s post hoc (*p* < 0.05).

For the dynamic conditions, we performed the Mann–Whitney U test for independent samples to verify non-significant differences in the sample characteristics. We conducted Friedman’s ANOVA to test the effect of time and group on the results. Bonferroni corrections were applied in order to limit the Type I error. All results were considered statistically significant when *p* ≤ 0.05.

### 2.8. Ethical Considerations

The children’s parents or legal guardians signed an informed consent form during an informative session prior to the start of this study. During the session, the researchers provided information about the data they were going to gather and analyze. This study was approved by the University’s Institutional Review Board (CD/50/2019). All the researchers followed the ethical guidelines established in the Declaration of Helsinki, 2008 edition. In order to counteract ethical issues, the families of the participating children could decide whether to be included in the experimental group or the control group, so that everyone had the opportunity to take part in the program. Likewise, the following academic year, a similar SL PE program was repeated without restricting access.

## 3. Results

This study compares the results obtained regarding the changes in static and dynamic postural control of ASD children who undertook a 6-month SL program with data gathered before and after the intervention; these data are also compared to the data gathered from a group of ASD children who did not participate in such a program.

When some constraints were added for the modification of visual, vestibular and proprioceptive pathways, it was observed that the experimental group showed statistically significant improvements in the vestibular pathway via *p* = 0.016) (Table 2).

The experimental group also showed better results in the EO_QS and EC_QS conditions, despite these results not showing significant differences. Meanwhile, the ASDC group showed better results in the EC_QS condition in both pre and post conditions after the 6-month period (Table 3).

We also observed improvements in the experimental group for the EO_RL and EC_LL conditions. Although these differences were not significant, we observed that they showed better results (Table 4).

The results of the dynamic test showed significant improvements in backward MXE and MV (*p* < 0.05) as well as in right and left DCL (*p* < 0.05) (Table 5).

Also, the results from the experimental group showed significant improvements in the AP condition of the EO_FS (Table 6).

## 4. Discussion

This study aimed to analyze the changes experienced by children with ASD who undertook a 6-month PE program two days a week using the SL methodology. We hypothesized that (1) the children who were involved in the program would significantly improve their static and dynamic postural control, (2) the experimental group would show significantly higher velocities in comparison to the control group regarding altered motor control, and (3) the participants in the intervention group would show better results in terms of vestibular, visual and somatosensory pathways after the program.

Results show several interesting findings. On the one hand, the first hypothesis was partially confirmed, since the results were statistically significant for dynamic postural control, but despite displaying improvements, the EO_FS and EC_FS conditions for static postural control did not achieve significant differences. On the other hand, with respect to the second hypothesis, we observed greater improvements in ASD_E_ than in ASD_C_ regarding increased velocity and better stability in postures such as standing, which is in line with the findings reported by Cheldavi et al. [34]. Lastly, in relation to the third hypothesis, all pathways reported an improvement after the intervention program for ASD_E_.

Regarding the evolution of postural control in the ASD children who participated in the SL program, our results are consistent with those of Kim et al. [35]. Despite their intervention for ASD children focusing on Taekwondo only being eight weeks long, they found improvements in single-leg stance balance with eyes closed and in double-leg stance balance on an unstable surface with eyes closed. In addition, these authors found that both their experimental and control groups improved in terms of their overall balance; however, they highlight that the results of particular components and conditions were slightly better for the experimental group, which is consistent with the results of the present study only for the EO_FS and EC_FS conditions.

For their part, Sarabzadeh et al. [43] implemented a 6-week Tai Chi Chuan program for ASD children. According to their results, there was a significant difference in balance variables between the pre-test and post-test scores comparing experimental and control groups. However, it is interesting to note that their control group outcomes were in a negative direction. In addition, they used a test instead of force platforms.

In any case, it must be considered that the two aforementioned studies used martial arts programs, which are sports that tend to be closely linked to balance [47,51,52,53], whereas the SL program carried out in the present study was approached from a general PE perspective aimed at developing a range of motor skills.

Results of the present study reported significant improvements in the AP direction for static balance outcomes. The study by Giagazoglou et al. [44], who analyzed the effects of a trampoline exercise intervention on the balancing ability of children with intellectual disabilities, also shows statistically significant improvements for the experimental group in this direction, particularly in double-leg stance with opened eyes, double-leg stance with closed eyes and one-leg stance. However, they also found significant differences in other outcomes in the ML direction. Focusing on ASD children, the research by Lidstone et al. [54] pointed to the ASD group showing increased AP sway magnitude and velocity compared to TD children, whereas ML sway findings displayed deficits. In this sense, differences between AP and ML sway directions have been previously reported [55,56], suggesting that they are related to central visual field integration deficits for postural control.

In this line, when focusing particularly on the evolution of static balance comparing control and experimental groups in our study, there was an overall improvement in both, but better results were displayed by the ASD_E_ group in the EO_FS and EC_FS conditions, which means that there was an improvement in vestibular and visual afferent inputs for postural control [57]. It should be noted that “paradoxical stress response” is a characteristic of some children with ASD [58], which means that they can show better stability results in the most difficult positions, such as EO_FS and EC_FS, which are the ones that improved in our study. Thus, when conditions are more difficult, children might have to pay more attention to the task, as opposed to when conditions were stable, when, to our understanding, children could become more distracted.

In the present study, we only observed statistically significant differences between pre and post-test measurements in ASD_E_ in the dynamic outcomes, for the conditions of LOS and MV backward movements (*p* < 0.05) and DCL right and left movements (*p* < 0.05). In contrast to these results, the study by Wang et al. [30] found that deficits in postural orientation and equilibrium were more pronounced during dynamic stances compared to static stances at a baseline. However, they suggest that increased demands through activities in which children must dynamically shift their CoP may have an effect on ordinary postural control deficits. Therefore, our findings may be related to the characteristics of the program developed, during which all participants took part in games and played in stable and unstable conditions, challenging their postural control systems in a dynamic way.

Regarding the third hypothesis and the afferent pathways involved, we observed that ASD children relied more on the vestibular pathway, followed by visual and somatosensory, which is in line with the results reported by Fong et al. [47]. The results for all pathways were improved after the intervention program for ASD_E_. However, these differences were not significant. In the study by Fong et al. [47], the authors observed significant differences in the visual and vestibular pathways after a taekwondo intervention program. Those findings were explained by the specificity of the exercises performed, which especially stimulated the sensory and vestibular functions. This is something that differs in our study, as we conducted PE activities aimed at improving the general motor proficiency of children with ASD.

Finally, according to the results obtained, it is suggested that participation in an SL PE program aimed at improving general motor proficiency seems to be a reliable way to develop the postural control of ASD children. In addition, this type of intervention usually represents an opportunity for children with ASD to increase their social and general motor skills [11,12,25], areas which are also affected by the characteristics of ASD. Consequently, it is important to provide ASD children with appropriate programs in order to guarantee their optimal development, avoid sedentary behavior and improve their quality of life and well-being [24,26]. In addition, this is particularly relevant since ASD children have fewer opportunities to participate in extracurricular exercise activities [24,46].

## 5. Conclusions

This study presents a few limitations that should be taken into account when interpreting the results. For example, the sample size was small; therefore, general and universal conclusions cannot be presented. However, studies with this type of population usually rely on data gathered with a similar number of participants [22,24,34,35,45]. In addition, small sample sizes are also common in SL research attempting to delve in and deeply understand the programs applied [59,60,61]. This is a limitation that usually comes with quasi-experimental designs using intervention programs. In addition, group formation (experimental and control) was not randomized but voluntary for two reasons: (1) to counteract ethical issues related to denying participation in a program that presumably came with benefits for participants, and (2) to ensure the viability of the study, since children belonging to the control group might not have been able to participate in the intervention program for different reasons (e.g., distance from the activity).

According to the results, the ASD children who participated in the 6-month SL PE program showed improved postural control. In addition, there were significant differences in the dynamic tests, probably because of the characteristics of the activities carried out during the intervention program. Training seems to be helpful to reduce motor limitations, and these benefits can be transferred to the daily life of children with ASD. However, more longitudinal programs are needed to observe other significant positive improvements.

To conclude, it is important to mention that this study has addressed the effects of an SL program on service receivers to fill a gap highlighted by previous literature [6,7]. In addition, results support the idea of SL emerging as a methodology capable of promoting the United Nations’ SDGs [62]. Particularly, the SL developed in this study has promoted the third objective concerned with the health and well-being of ASD children while enhancing the quality education of pre-service teachers and their future teaching practices.

## Figures and Tables

**Table 1:** Modified afferent inputs according to the different tests (adapted from Molloy et al., 2003 [36]).

Acronym	Test	Afferent Input
EO_QS	Feet on platform, eyes open	Visual, somatosensory, vestibular
EC_QS	Feet on platform, eyes closed	Somatosensory, vestibular
EO_FS	Feet on foam, eyes open	Visual, vestibular, modified somatosensory
EC_FS	Feet on foam, eyes closed	Vestibular, modified somatosensory

**Table 2:** Comparison of afferent inputs and group × time effect.

	ASD_E_		ASD_C_		Group × Time Effect
	Pre	Post	Pre	Post	
	(M ± SD)	(M ± SD)	(M ± SD)	(M ± SD)	*p*-Value
Somatosensorial	1.41 ± 0.63	2.06 ± 2.38	1.08 ± 0.56	1.17 ± 0.43	*p* = 0.204
Visual	2.07 ± 0.79	2.79 ± 1.54	1.93 ± 0.75	1.95 ± 0.89	*p* = 0.115
Vestibular	3.68 ± 2.37	4.96 ± 2.76 **	5.09 ± 5.01	5.15 ± 4.68	*p* = 0.016

**Table 3:** Descriptive and statistical values for the CoP during EO_QS, EC_QS, EO_FS and EC_FS conditions by the ASD_E_ and ASD_C_ groups.

	ASD_E_					ASD_C_				
	Pre	Post				Pre	Post			
Stability Quotient	Gra/s (M ± SD)	Gra/s (M ± SD)	IC_95%_	*p-Value*	*Cohen’s d*	Gra/s (M ± SD)	Gra/s (M ± SD)	IC_95%_	*p-Value*	*Cohen’s d*
EO_QS	0.85 ± 0.45	0.76 ± 0.40	−0.152, 0.309	0.398	0.20	0.94 ± 0.88	1.18 ± 1.65	−0.951, 0.476	0.457	−0.12
EC_QS	3.12 ± 7.76	1.64 ± 2.66	−3.303, 5.989	0.493	0.19	0.71 ± 0.24	1.16 ± 1.44	−1.484, 0.584	0.338	−0.15
EO_FS	1.54 ± 0.71	1.76 ± 1.00	−0.785, 0.343	0.205	−0.23	1.39 ± 0.67	1.39 ± 0.76	−0.245, 0.245	1.000	−0.26
EC_FS	2.54 ± 1.22	2.99 ± 1.05	−0.938, 0.532	0.488	−0.52	2.73 ± 1.06	2.88 ± 1.45	−1.145, 0.845	0.732	−0.22

EO: eyes opened, EC: eyes closed, QS: quiet standing; FS: foam standing.

**Table 4:** Descriptive values (M ± SD) for the conditions of eyes opened and closed.

	ASD_E_					ASD_C_				
Age Group	Pre	Post	IC_95%_	*d*	*p*-Value	Pre	Post	IC_95%_	*d*	*p*-Value
Stability Quotient	º/s (M ± SD)	º/s (M ± SD)				º/s (M ± SD)	º/s (M ± SD)			
EO_RL	9.69 ± 2.84	9.66 ± 3.48	−1.257, 1.324	−0.009	0.957	8.2 ± 4.7	9.5 ± 3.8	−4.942, 2.292	0.304	0.415
EO_LL	11.76 ± 0.91	11.50 ± 1.27	−0.266, 0.780	−0.235	0.308	10.7 ± 3.7	12.0 ± 0.0	−4.374, 1.774	0.50	0.351
EC_RL	8.98 ± 3.70	8.04 ± 3.89	−0.728, 2.599	−0.248	0.246	9.2 ± 3.6	9.4 ± 3.0	−4.469, 3.969	0.060	0.893
EC_LL	11.28 ± 1.43	11.74 ± 0.96	−1.526, 0.597	0.186	0.362	12.0 ± 0.0	12.0 ± 0.0	0, 0	0	1.000

**Table 5:** Results of backward MXE and MV.

		Reaction Time (s)			LOS (%)			MV (º/s)			DCL (%)		
		Pre (Range)	Post (Range)	F, *p*-Value	Pre (Range)	Post (Range)	F, *p*-Value	Pre (Range)	Post (Range)	F, *p*-Value	Pre (Range)	Post (Range)	F, *p*-Value
Forward	E C	0.80 (0.70–1.12) 1.17 (1.01–1.35)	0.90 (0.31–1.19) 0.95 (0.41–1.18)	F = 0.99 *p* = 0.40	64.00 (36.00–88.00) 78.00 (48.00– 89.00)	78.00 (55.00–85.00) 72.00 (31.00–98.00)	F = 0.94 *p* = 0.43	4.00 (1.40–8.50) 3.50 (2.30–5.60)	3.50 (3.10–7.00) 4.90 (2.20–6.10)	F = 2.28 *p* = 0.09	67.00 (65.00–81.00) 80.00 (34.00–86.00)	73.00 (49.00–83.00) 63.00 (47.00–83.00)	F = 2.30 *p* = 0.09
Right Forward	E C	0.70 (0.58–1.22) 0.99 (0.39–1.20)	0.68 (0.62–1.38) 1.1 (0.53–1.24)	F = 0.69 *p* = 0.56	73.00 (46.00–95.00) 84.00 (60.00–111.00)	63.00 (55.00–95.00) 85.00 (75.00–89.00)	F = 1.90 *p* = 0.14	4.90 (3.10–9.30) 6.20 (4.10–7.80)	5.10 (3.15–7.65) 5.10 (4.50–6.10)	F = 0.62 *p* = 0.60	43.00 (18.00–65.00) 54.00 (28.00–74.00)	40.00 (5.00–64.00) 60.00 (48.00–64.00)	F = 4.35 *p* = 0.10
Right	E C	0.79 (0.70–1.03) 0.79 (0.55–1.19)	0.81 (0.66–1.17) 0.75 (0.56–0.83)	F = 1.72 *p* = 0.18	79.50 (57.25–87.25) 88.00 (62.00–97.00)	78.00 (72.00–87.00) 72.00 (55.00–85.00)	F = 2.71 *p* = 0.05	5.35 (2.40–7.18) 6.40 (5.20–7.40)	6.40 (5.10–10.10) 6.80 (6.60–7.40)	F = 2.38 *p* = 0.08	70.50 (48.00–77.75) 77.00 (70.00–82.00) *	73.00 (54.00–81.00) 72.00 (58.00–74.00) *	F = 3.03 *p* = 0.04
Right Backward	E C	0.95 (0.35–1.02) 0.56 (0.35–1.27)	0.81 (0.49–0.98) 0.85 (0.72–0.98)	F = 1.03 *p* = 0.39	67.00 (48.00–88.00) 54.00 (49.00–75.00)	86.00 (51.00–101.00) 73.00 (54.00–90.00)	F = 0.95 *p* = 0.42	4.80 (2.30–5.50) 3.5 (3.10–6.20)	4.30 (3.30–6.50) 3.70 (3.40–6.00)	F = 0.57 *p* = 0.64	50.00 (17.00–79.00) 31.00 (5.00–61.00)	29.00 (10.00–62.00) 42.00 (0–56.00)	F = 0.11 *p* = 0.95
Backward	E C	0.71 (0.35–1.07) 0.87 (0.57–1.02)	0.75 (0.62–1.04) 0.81 (0.76–1.09)	F = 1.26 *p* = 0.30	63.50 (57.00–75.00) * 64.00 (54.00–94.00)	70.00 (56.00–92.00) * 65.00 (59.00–85.00)	F = 0.30 *p* = 0.82	2.10 (1.63–3.73) * 2.8 (2.20–3.10)	3.70 (2.80–4.70) * 3.30 (2.90–4.20)	F = 0.50 *p* = 0.69	56.00 (31.50–69.75) 57.00 (14.00–70.00)	60.00 (37.00–66.00) 58.00 (47.00–70.00)	F = 0.29 *p* = 0.83
Left Backward	E C	1.03 (0.70–1.62) 0.72 (0.50–1.42)	0.86 (0.48–1.10) 0.82 (0.60–1.22)	F = 2.05 *p* = 0.12	67.00 (37.50–89.50) 88.00 (59.00–92.00)	79.00 (64.00–99.00) 60.00 (57.00–65.00)	F = 4.38 *p* = 0.01	4.25 (2.68–4.80) 6.10 (4.80–6.90)	4.90 (3.40–6.70) 4.30 (2.90–5.80)	F = 0.25 *p* = 0.86	27.50 (14.50–58.00) 52.00 (33.00–59.00)	36.00 (7.00–56.00) 42.00 (31.00–57.00)	F = 0.89 *p* = 0.46
Left	E C	0.92 (0.73–1.08) 0.59 (0.55–0.81)	1.20 (0.65–1.73) 0.82 (0.60–1.22)	F = 1.02 *p* = 0.39	73.50 (51.00–86.00) 84.00 (73.00–88.00)	84.00 (71.00–86.75) 66.00 (58.00–88.00)	F = 2.55 *p* = 0.07	5.38 (2.60–8.03) 7.30 (6.20–9.00)	6.25 (3.75–9.68) 5.60 (5.00–6.70)	F = 2.53 *p* = 0.07	63.00 (48.25–73.75) 73.00 (71.00–87.00) *	70.00 (54.25–81.75) 63.00 (53.00–81.00) *	F = 2.94 *p* = 0.04
Left Forward	E C	0.96 (0.61–1.39) 0.98 (0.59–1.33)	0.85 (0.62–1.57) 0.73 (0.68–1.07)	F = 2.51 *p* = 0.07	58.00 (45.50–91.50) 76.00 (64.00–83.00)	71.5 (61.00–78.75) 81.00 (53.00–99.00)	F = 1.46 *p* = 0.24	5.10 (2.18–7.68) 6.10 (5.60–7.30)	3.80 (2.70–8.13) 7.90 (5.70–10.30)	F = 1.70 *p* = 0.18	58.00 (32.00–65.25) 62.00 (51.00–78.00)	48.5 (22.00–79.00) 74.00 (54.00–86.00)	F = 2.64 *p* = 0.06

*: *p* < 0.05.

**Table 6:** Descriptive and statistical values of the CoP in the AP and ML.

	ASD_E_			ASD_C_		
	Pre	Post		Pre	Post	
	(M ± SD)	(M ± SD)	*d*	(M ± SD)	(M ± SD)	*d*
CoP_EO_QS_ML	0.00 ± 0.86	0.10 ± 0.59	−0.07	0.34 ± 0.98	−0.30 ± 0.51	−0.27
CoP_EC_QS_ML	0.14 ± 0.97	−0.29 ± 0.68	0.06	0.12 ± 0.97	−0.99 ± 0.25	−0.36
CoP_EO_FS_ML	0.80 ± 0.67	0.55 ± 0.97	0.52	−0.11 ± 0.47	−0.23 ± 0.19	0
CoP_EC_FS_ML	0.43 ± 0.80	0.04 ± 0.99	0.26	−0.24 ± 0.38	0.10 ± 0.73	0
CoP_EO_QS_AP	−0.48 ± 0.89	−0.53 ± 0.93	0.40	−0.80 ± 1.13	−0.69 ± 2.80	−0.14
CoP_EC_QS_AP	−0.40 ± 0.67	−0.61 ± 0.78	0.64	−0.80 ± 0.99	−0.79 ± 0.18	−0.02
CoP_EO_FS_AP	0.63 ± 0.80 *	0.21 ± 0.99 *	0.38	0.99 ± 1.17	0.43 ± 0.42	−0.23
CoP_EC_FS_AP	0.38 ± 1.32	0.92 ± 1.20	−0.21	0.84 ± 0.99	0.90 ± 1.40	−0.22

*: *p* < 0.05.

## Data Availability

The data presented in this study is not available to preserve the participants privacy.
